# Ablation of Survivin in T Cells Attenuates Acute Allograft Rejection after Murine Heterotopic Heart Transplantation by Inducing Apoptosis

**DOI:** 10.3389/fimmu.2021.710904

**Published:** 2021-08-06

**Authors:** Heng Xu, Jizhang Yu, Jikai Cui, Zhang Chen, Xi Zhang, Yanqiang Zou, Yifan Du, Yuan Li, Sheng Le, Lang Jiang, Jiahong Xia, Jie Wu

**Affiliations:** Department of Cardiovascular Surgery, Union Hospital, Tongji Medical College, Huazhong University of Science and Technology, Wuhan, China

**Keywords:** transplantation immunology, survivin, *Birc5*, T cell, apoptosis

## Abstract

Although studies in oncology have well explored the pharmacological effects of *Birc5*, little is known about its role in allogeneic T-cell responses. Therefore, the present study used a mouse model of acute heart allograft rejection to investigate the protective effect and mechanism of conditional knockout of *Birc5* in T cells. Survivin (encoded by *Birc5*) was up-regulated in T cells activated *in vivo* and *in vitro*. Deletion of *Birc5* in T cells attenuated acute heart allograft rejection by reducing the ratio of effector to naive T cells and Th1 to Tregs. In addition, deletion of *Birc5* had no noticeable effect on proliferation but on apoptosis and the secretion of IFN-γ. The results revealed a significant increase in the percentage of Annexin V positive CD4^+^ T cells in the *Birc5^-/-^* group, compared to the WT. Moreover, there was significant increase in early apoptotic alloreactive T cells in *Birc5* ^-/-^ mice and this was partly mediated by caspase-3. Furthermore, treatment with YM155 inhibited acute heart allograft rejection *in vivo* and increased T-cell apoptosis in healthy human PBMCs *in vitro*. The results highlight a potential therapeutic target for the prevention and treatment of acute transplant rejection.

## Introduction

Heart transplantation is the most effective and essential clinical treatment for terminal refractory heart failure caused by various diseases (1–3). Although great progress has been made on controlling acute transplant rejection, the long term survival of patients is affected by such factors as the rejection-mediated graft vascular disease (which causes graft ischemia and functional failure) and the side effects caused by various immunosuppressive agents such as infection, renal fibrosis and malignant tumors (4, 5). Therefore, it is necessary to identify new targets in order to control transplant rejection or enhance immune tolerance. Given that T cells were proven to play an indispensable role in the occurrence of rejection after transplantation (6, 7), T cell survival and function has gained popularity in the field of transplantation immunology.

In addition*, Birc5* encodes the survivin protein which is a known inhibitor of apoptosis (8). Notably, survivin plays an essential role in mitosis by ensuring proper separation of chromosomes (9, 10) and also prevents apoptosis by hindering the activation of the caspase pathway (11). Moreover, survivin is used as a biomarker of resistance to chemotherapy, increased metastatic activity and risk of tumor recurrence, in cancer therapy (12). Survivin also plays an essential role in the maturation and proliferation of T cells (13) and in regulating T cell responses (14, 15). Additionally, upregulated levels of survivin was shown to affect the viability and responses of infected CD4^+^ T cells, in antiviral immunity (14). Furthermore, silencing the *Birc5* gene leads to enhanced apoptosis as well as reduced viability and proliferation of inflammatory T cells in autoimmune disease (16, 17). Nonetheless, little is known about the role of survivin in transplantation immune responses.

Consequently, the preset study demonstrated that deletion of *Birc5* in T cells attenuates acute heart allograft rejection, which is associated with decreased alloreactive T cell responses and enhanced T cell apoptosis in a caspase-3-dependent manner. Moreover, a mouse model was used to show that treatment with inhibitor, YM155 can also attenuate acute heart allograft rejection. The results therefore showed that survivin may be a new drug target for use in the treatment of acute transplant rejection.

## Materials and Methods

### Animals

The B6.129P2-*Birc5^tm1Mak^*/J (*Birc5^flox/flox^*) and *CD4-Cre* mice were obtained from the Jackson Laboratories (Bar Harbor, ME) while the C57BL/6 and BALB/c mice were purchased from the Shanghai Model Organisms Center (Shanghai, China). Thereafter, the *Birc5^flox/flox^* mice were crossed with the *CD4-Cre* mice to generate the *Birc5^flox/flox^CD4-Cre* mice (*Birc5^-/-^*). All the mice were bred under specific pathogen-free conditions in the Laboratory Animal Center, Huazhong University of Science and Technology (Wuhan, China). Moreover, male mice that were 8 to 10 weeks old were used in the subsequent experiments. All the animal experiments were approved by the Animal Care and Use Committee of Tongji Medical College (Wuhan, China).

### The Heterotopic Heart Transplantation Model

The heterotopic cardiac transplantation model was generated as previously reported (18) and allograft function was monitored every day through palpation. Briefly, hearts were obtained from BALB/C mice then stitched to the aorta abdominalis and postcava of C57BL/6 mice. The mice were divided into three groups, i.e., the isograft group and 2 allograft categories. The isograft group received the C57BL/6 heart then treated with saline while the other two allograft categories received intraperitoneal (i.p.) injection of either saline or YM155 (5 mg/kg) on day 1, 3 and 5 after the operation. Additionally, the spleen or grafts from recipient mice were extracted on the 6^th^ day for analysis.

### Isolation of Heart-Infiltrating CD45 Cells

The heart-infiltrating cells were isolated, as previously described (18). Briefly, the heart tissues were obtained and cut into pieces after which the tissue fragments were digested with 1 mg/mL of collagenase B (Roche 11088815001) or Collagenase Type 2 (Sangon Biotech A004174-0100) in HEPES buffer then rotated gently for 45 min at 37°C. In addition, cell suspensions were filtered with 70-μm cell strainers (BIOFIL) and Percoll (Beijing Solarbio Science & Technology Co., Ltd.) was used to purify heart mononuclear cells through density centrifugation.

### *In Vitro* T Cell Sorting, Activation, and the CTV Labelled Proliferative Assay

Naive CD4^+^ T cells were isolated from WT B6, *Birc5^flox/flox^CD4-Cre* or *Birc5^flox/flox^* mice through magnetic bead sorting of CD3^+^CD4^+^CD25^-^ T cells using the LS columns (Miltenyi). Purified CD4^+^ T cells (2×10^5^ cells/well) were then plated in 96-well plates that had been pre-coated with 5 μg/mL of anti-CD3 Ab (BioLegend) for 4 hours, after which soluble anti-CD28 Ab (BioLegend) was added (final concentration, 1 μg/mL). Afterwards, T cells were cultured and treated as described in each experiment then finally collected for analysis. In order to evaluate the effect of *Birc5* knockout on the proliferative potential of CD4 T cells *in vitro*, the study labelled the cells using the CellTrace Violet (CTV) fluorochrome then stimulated them in 96-well plates coated with the anti-CD3 Ab and soluble anti-CD28 Ab. The rest of the experiments including Mixed-lymphocyte Reactions (MLR) (19), western blotting (20), FCM and ELISA (21) are included in the supplemental experimental procedures.

## Results

### Survivin Was Upregulated in Allograft-Infiltrated T Cells During Acute Allograft Rejection

**Figure 1:**
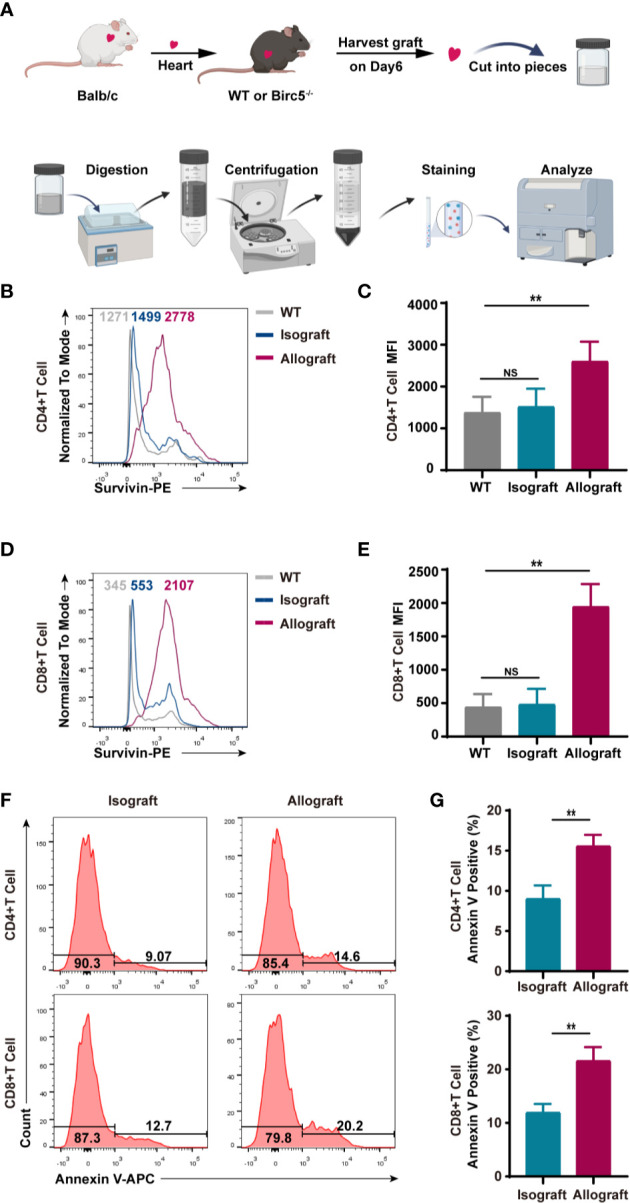
T cells in murine heart allografts had high expression of survivin and apoptosis compared to those in the isograft group. **(A)** A schematic of the experimental design and procedure of isolating heart infiltrating lymphocytes. **(B–D)** Representative histogram plots of survivin. **(C, E)** Bar graphs of the MFI (mean fluorescence intensity) of CD4^+^ and CD8^+^ T cells. T cells were isolated from the isograft or allograft mice, 6 days after transplantation. **(F)** A representative histogram of Annexin V staining of CD4^+^ and CD8^+^ T cells derived from infiltrated CD45+ cells in isograft and allograft mice. **(G)** FCM analysis of the percentage of Annexin V positive CD4^+^ and CD8^+^ T cells in isograft and allograft mice. Data are representative of 3 independent experiments (n = 4-6 mice per group). Bars represent the mean ± SD. **p < 0.01, ns, not statistically significant.

Based on the procedure in **Figure 1A**, the study separated the infiltrated CD45^+^ cells on the 6^th^ day after heart transplantation then assessed the levels of survivin in alloreactive T cells. The FCM histogram plots demonstrated that survivin was upregulated in alloreactive CD4^+^ and CD8^+^ T cells (**Figures 1B–E**), in the heart. Additionally, the study used live/dead staining to gate out the dead cells and in the next gate the Annexin V positive cells were considered as early apoptotic cells. Thereafter, flow cytometry was used to examine the levels of early apoptotic alloreactive T cells. Interestingly, the results showed that the percentage of Annexin V positive alloreactive T cells was higher than that in the isograft group (**Figures 1F, G**).

### Conditional Knockout *Birc5* in T Cells Alleviates Acute Allograft Rejection Following Murine Heterotopic Heart Transplantation

**Figure 2:**
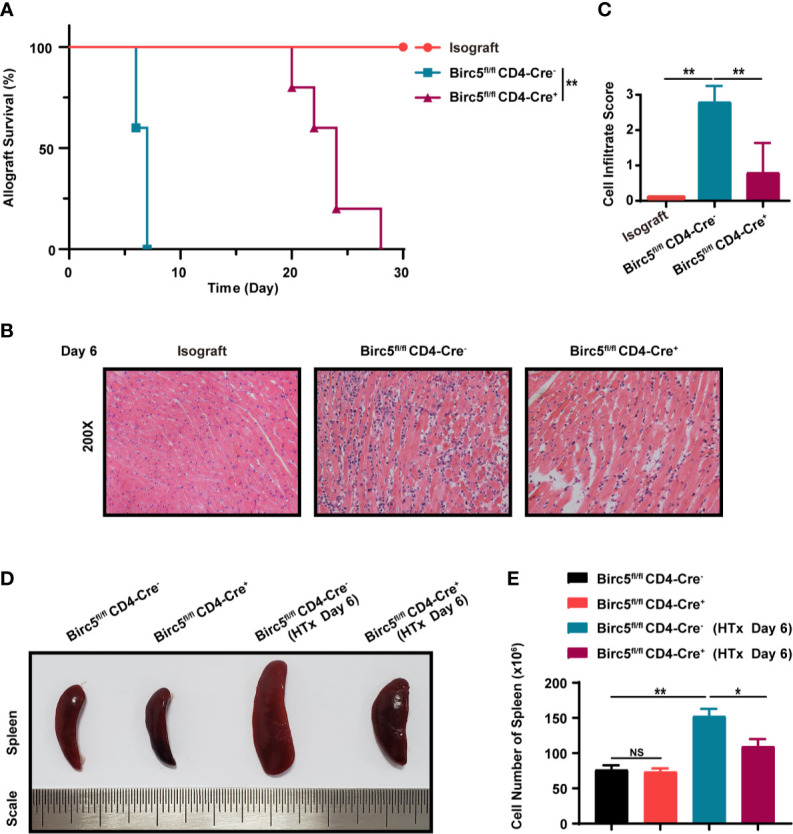
Conditional knockout of *Birc5* in T cells alleviates acute allograft rejection after murine heterotopic heart transplantation. **(A)** Kaplan-Meier survival curves of heart allografts in Isograft, WT and *Birc5^-/-^* recipients. **(B)** Representative H&E staining results showing infiltration of immune cells in grafts in Isograft, WT and *Birc5^-/-^* recipients. **(C)** Histological analysis of the immune cell infiltration score in the heart. **(D)** Representative appearance of the spleen in the four groups and **(E)** quantification of cells in the spleen of each group. Data are representative of 3 independent experiments (n = 5 mice per group). Bars represent the mean ± SD. *p < 0.05, **p < 0.01, ns, not statistically significant, log-rank (Mantel-Cox) test **(A)**, Wilcoxon’s and Student’s t test (2 groups).

In order to verify the effect of survivin on the rejection of cardiac transplants, BALB/c hearts were transplanted into *Birc5^flox/flox^CD4-Cre^+^* (*Birc5^-/-^*) or *Birc5^flox/flox^ CD4-Cre^-^* (WT) mice. The results showed that the Mean Survival Time (MST) of the grafts was longer in *Birc5^-/-^* recipients than in the control group (**Figure 2A**). Additionally, H&E staining of the heart grafts on the 6^th^ day after transplantation showed that mice in the *Birc5^-/-^* group had lower heart inflammatory cell infiltration scores compared to the control category (**Figures 2B, C**). Moreover, the spleen of mice in the *Birc5^-/-^* group had no evident splenomegaly. After grinding, filtration and red blood cell lysis using ACK lysing buffer, we calculated the cell number in the spleen. There was also a remarkable decrease in the number of spleen cells of mice in the *Birc5^-/-^* group compared to those in the control category (**Figures 2D, E**). The percentage and absolute cell numbers of CD4^+^ T cells and CD8^+^ T cells in spleen from indicated groups was shown in **Supplemental Figures 3D, E**.

### The Effect Knocking Out Survivin on the Subsets of T Cells in the Spleen After Heart Transplantation

**Figure 3:**
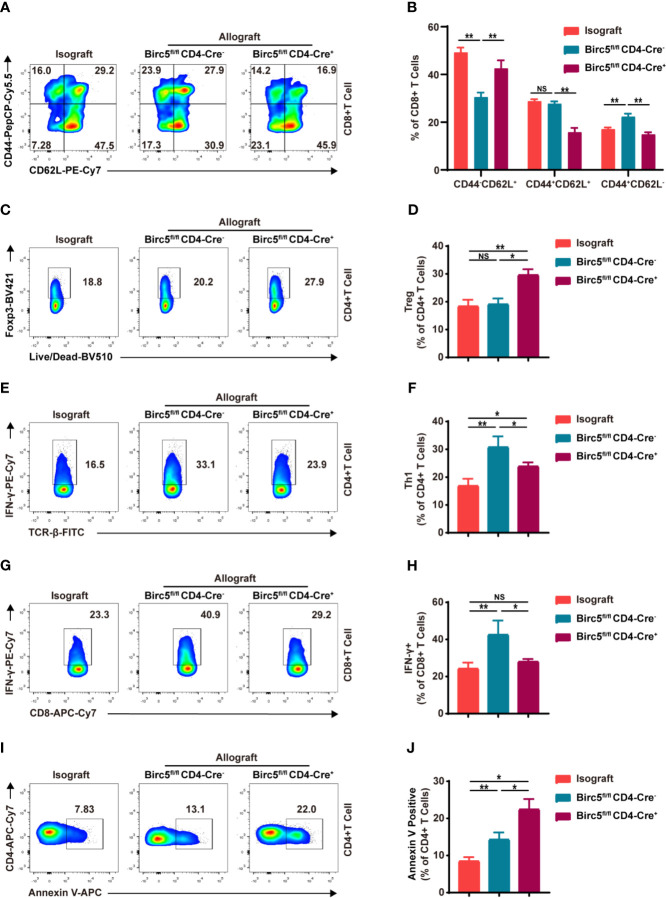
Impact of *Birc5* knockout on the subsets of T cells in the spleen after heart transplantation. **(A)** Representative flow plots of the expression of CD44 and CD62L on CD8^+^ T cells. **(B)** Bar graphs represent the percentages ± SD of each gated sub-population of naive (CD62L^+^CD44^–^), effector/effector memory (CD62L^–^CD44^+^) and central memory (CD62L^+^CD44^+^) T cells on CD8^+^ T cells from indicated groups. **(C–H)** Representative flow plots of **(C)** CD4^+^Foxp3^+^, **(E)** CD4^+^IFN-γ^+^ and **(G)** CD8^+^IFN-γ^+^. **(D, F, H)** Percentage of the parent gated cells in mice in the isograft, allograft WT or *Birc5* ^-/-^ groups. **(I)** Flow-cytometry plots and **(J)** summary graph of Annexin V^+^ live/dead^-^ (early apoptosis) T cells isolated from the spleen of WT or *Birc5*-/- mice at 6-8 weeks of age. Flow cytometry analysis of cytokine production by CD4^+^ and CD8^+^ T cells after re-stimulation with PMA and ionomycin in the presence of Golgi-Stop. Data are representative of 3 independent experiments (n = 5 mice per group). Bars represent mean ± SD, *p < 0.05, **p < 0.01, ns, not statistically significant. See also **Supplemental Figure 3**.

The study then analyzed the effects of *Birc5* knockout on T-cell subpopulations in the spleen after heart transplantation. Therefore, the spleen was harvested on the 6^th^ day after transplantation then assessed through flow cytometry. The gating strategy in the spleen is shown in **Supplemental Figure 3A** as previous studies (22–24). The findings revealed a significant decrease in the proportion of effector/effector memory (CD62L^-^CD44^+^) T cells in the *Birc5^-/-^* group. Meanwhile, the proportion of naive (CD62L^+^CD44^–^) T cells was increased (**Figures 3A, B**). The CD4^+^ T graph is shown in **Supplemental Figures 3B, C**. In addition, the proportion of CD4^+^Foxp3^+^ T cells significantly increased in *Birc5^-/-^* recipients although there was a decrease in CD8^+^IFN-γ^+^ and CD4^+^IFN-γ^+^ T cells (**Figures 3C–H**). The percentage of CD4^+^ T cells and CD8^+^ T cells in spleen from indicated groups was shown in **Supplemental Figure 3D**. The absolute cell numbers relative was shown in **Supplemental Figures 3E, F**. Moreover, the flow cytometry plots showed that the spleen of *Birc5* ^-/-^ mice had a higher proportion of Annexin V^+^ apoptotic CD4+ T cells that than the WT group (**Figures 3I, J**).

### Knock-Out of *Birc5* INDUCED MORE APOPTOSIS of T Cells and Had No Obvious Effect on Proliferation

**Figure 4:**
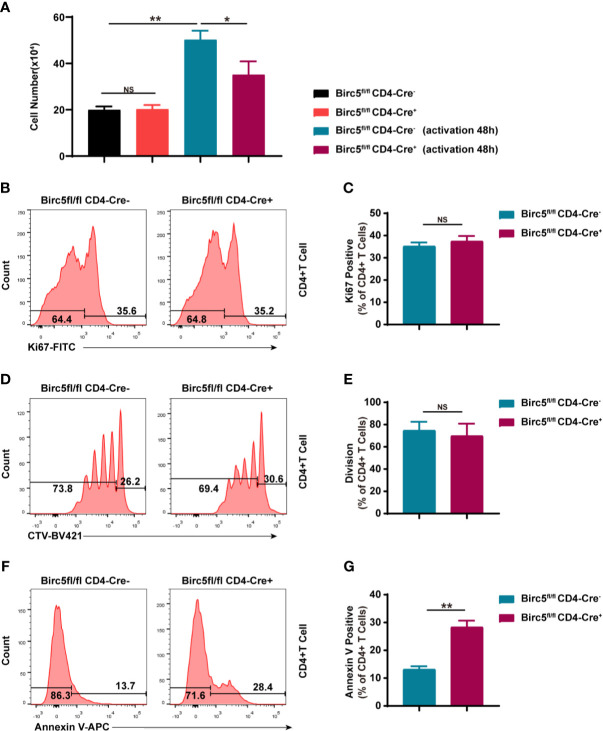
*In vitro* knockout of *Birc5* in T cells causes a decrease in the number of T cells through apoptosis and not by interfering with proliferation. **(A)** The number of CD4^+^ T cells isolated from the spleen of WT or *Birc5* ^-/-^ mice before and after activation *in vitro*. Representative histogram plots of **(B)** Ki67, **(D)** CTV and **(F)** Annexin V positive cells. **(C, E, G)** Bar graphs of the percentage of parent gated T cells isolated from the spleen of WT or *Birc5* ^-/-^ mice at 6-8 weeks of age following stimulation with anti-CD3/CD28 for 48h *in vitro*. Data are representative of 4 independent experiments (n = 3 mice per group). Data is presented as the mean ± SD. *p < 0.05, **p < 0.01, ns, not statistically significant.

The study then cultured the same number of CD4^+^ T cells from both *Birc5^-/-^* and the WT mice, with or without activation. 48 hours later, the number of CD4^+^ T cells in *Birc5^-/-^* mice was less than that in the control group (**Figure 4A**). The western blotting demonstrated that survivin was upregulated in T cells after activation (**Supplemental Figures 2A, B**). Knock-out of *Birc5* could down-regulate the survivin expression (**Supplemental Figures 2C, D**). In addition, the study examined whether the reduced number of CD4^+^ T cells was due to decrease in proliferation or apoptosis. The results revealed no significant differences in the levels of the proliferation marker, Ki67 between the two groups (**Figures 4B, C**). Moreover, naive CD4^+^ T cells were labelled with CTV then the proliferative assay was conducted. The findings showed similar levels of the proliferation in CD4^+^ T cells following the dilution of CTV (**Figure 4D, E**). On the contrary, the Birc5^-/-^ mice had a higher percentage of Annexin V^+^ apoptotic T cells (**Figure 4F, G**).

### The Apoptosis Induced by *Birc5* Knockout in T Cells Was Partly Dependent on Caspase3 Activation

**Figure 5:**
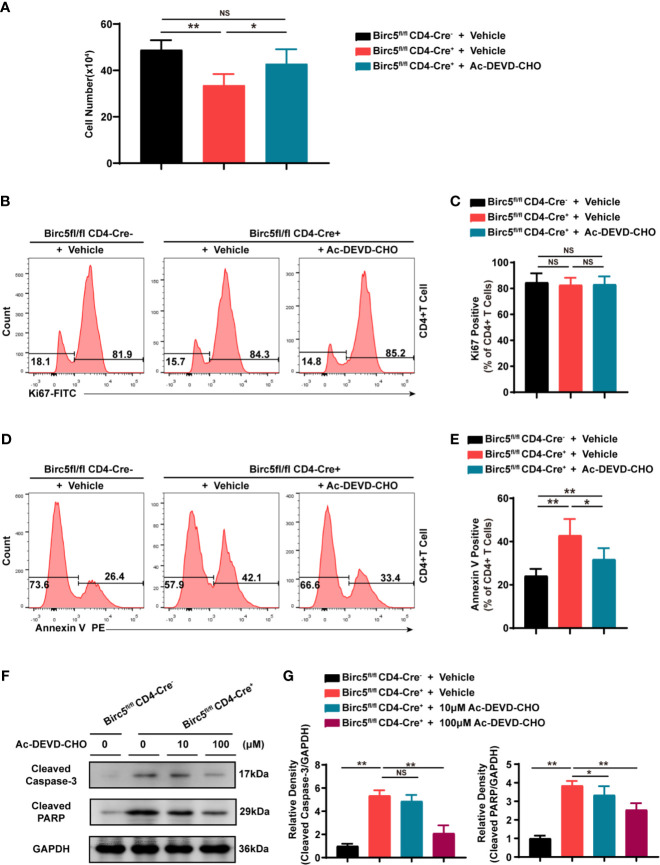
The apoptosis induced by *Birc5* knockout in T cells was partly dependent on caspase3. **(A)** The number of CD4^+^ T cells isolated from the spleen of WT or *Birc5* ^-/-^ mice before and after activation *in vitro*. **(B–E)** Representative histogram plots of **(B)** Ki67 and **(D)** Annexin V positive cells. **(C, E)** Bar graphs indicating the percentage of the parent gated T cells isolated from the spleen of WT or *Birc5* ^-/-^ mice at 6-8 weeks of age following stimulation with anti-CD3/CD28 for 48h *in vitro*. **(F)** The expression of Cleaved-CASP3 and Cleaved-PARP1 was examined through western blotting after isolating T cells from the spleen of WT or *Birc5^-/-^* mice. The T cells were stimulated *in vitro* with anti-CD3 Abs for 2 days then treated in the absence or presence of Ac-DEVD-CHO for 24h. GAPDH was used as an internal control. **(G)** Quantitative data of the groups indicated in **(F)**. Data are representative of 3 independent experiments (n = 5 per group). The OD values in column graphs are presented as means ± SD, *p < 0.05, **p < 0.01, ns, not statistically significant. See also **Supplemental Figure 2**.

Thereafter, the study cultured the same number of CD4^+^ T cells from Birc5^-/-^ and WT mice for 48 hours, in order to examine the underlying mechanism of apoptosis induced by *Birc5* knockout. The results showed that the number of CD4^+^ T cells from Birc5^-/-^ mice was less than that in the control group. However, the number increased after treatment with the Caspase 3 inhibitor, Ac-DEVD-CHO (**Figure 5A**). The study then assessed whether the decrease in the number of CD4^+^ T cells was due to proliferation or apoptosis. Analysis of the proliferation marker, Ki67 revealed no significant difference between the two groups (**Figure 5B, C**). However, the proportion of Annexin V^+^ apoptotic cells T cells was higher in Birc5^-/-^ mice than in the controls. Additionally, treatment with Ac-DEVD-CHO led to a decrease in the proportion of apoptotic cells (**Figures 5D, E**). These results therefore suggested that Ac-DEVD-CHO restored the number of cells and decreased the percentage of apoptotic cells without affecting the ratio of ki67^+^ cells. Furthermore, the levels of Cleaved-CASP3 and Cleaved-PARP1 were assessed in the stimulated CD4^+^ T cells. The results revealed significant upregulation of Cleaved-CASP3 and Cleaved-PARP1 in survivin deficient CD4^+^ T cells, compared to the controls. However, treatment with Ac-DEVD-CHO restored the levels of both Cleaved-CASP3 and Cleaved-PARP1 (**Figures 5F, G**).

### YM155 Suppresses Acute Allograft Rejection Following Murine Heterotopic Heart Transplantation

**Figure 6:**
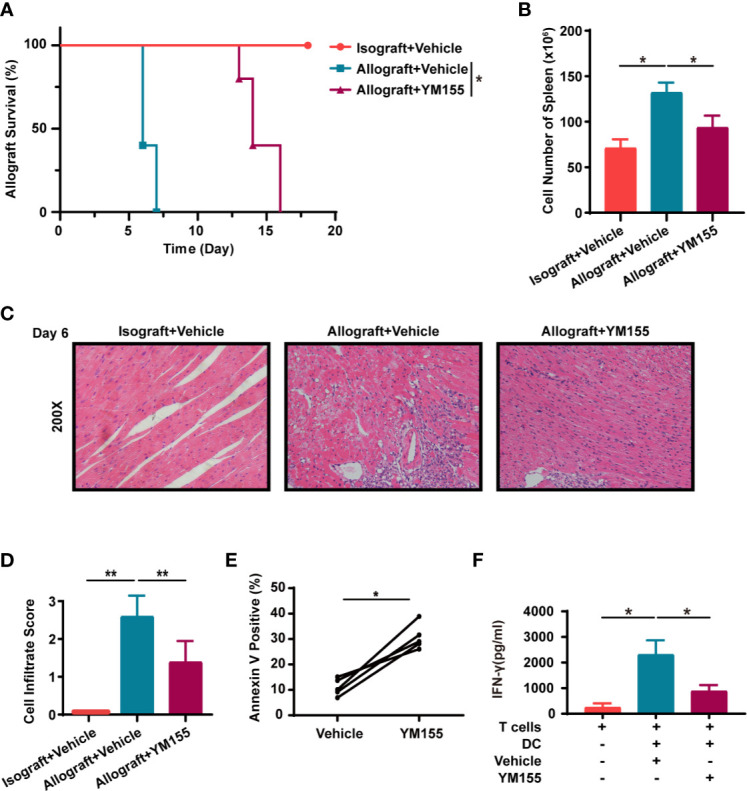
YM155 suppresses acute allograft rejection after murine heterotopic heart transplantation and reduces the secretion of IFN-γ in human T lymphocytes. **(A)** Kaplan-Meier survival curves of the heart allografts in the isograft, vehicle or YM155 group. **(B)** Cell count in the spleen of mice in the isograft, vehicle or YM155 groups. **(C)** Representative H&E staining results showing infiltration of cells in grafts in the isograft, vehicle or YM155 group. **(D)** Histological analysis of **(C)** the heart cell infiltrate score. **(E)** YM155 similarly increased apoptosis in human PBMC T cells after activation *in vitro*. Healthy-donor PBMCs were stimulated with anti-CD3 and anti-CD28 Abs for 2 days then treated with YM155 for 24 h at 100 nM on day 3. **(F)** Concentration of IFN-γ in the supernatant of MLR, assessed through ELISA. The histogram shows the concentration of IFN-γ after different treatments. Data are representative of 3 independent experiments in **(A–D)**. Data is presented as the mean ± SD of one representative experiment out of the five conducted in **(E, F)** [*p < 0.05, **p < 0.01, n = 5 per group, log-rank (Mantel-Cox) test **(A)**, Wilcoxon’s and Student’s t test (2 groups)]. See also **Supplemental Figure 1**.

YM155 is a small molecule that is known to inhibit survivin. Notably, YM155 can selectively inhibit the expression of survivin at both the mRNA and protein levels. YM155 was also shown to have anticancer activity in many malignancies (25). In this study, survivin was up-regulated when T cells were in the activation stage but was down-regulated after treatment with YM155 (**Supplemental Figures 2E, F**). In addition, YM155 increased the apoptosis of T cells *in vitro* although treatment with Ac-DEVD-CHO reversed this effect (**Supplemental Figures 1A, B**). Thereafter, the study choose a safe and effective dose of YM155 as reported in previous research, for animal experiments (26, 27). Mice in the isograft group were treated with the vehicle (PBS) while those in the allograft group received either YM155 (5mg/kg on -1, 1, 3, 5 days) or the vehicle through intraperitoneal injection. The Mean Survival Time (MST) of grafts in mice treated with YM155 was 14 days which was 8 days longer than that in the vehicle group (n = 5; *p* < 0.01; **Figure 6A**). Additionally, there was a significant decrease in the number of splenocytes in mice treated with YM155, on the 6^th^ day (**Figure 6B**). Moreover, H&E staining of the grafts showed that the YM155 group had cell infiltration scores lower than the vehicle category (2.40 ± 0.54 *vs.* 1.00 ± 0.70; **Figures 6C, D**).

### YM155 Reduces the Secretion of IFN-γ in a Human Mixed Lymphocyte Reaction

Similarly, YM155 increased the apoptosis of human PBMC T cells *in vitro* (**Figure 6E**). In order to evaluate the effect of YM155 on the secretion of inflammatory factors from T cells, human MLR was conducted and the supernatant was collected on the 5^th^ day for ELISA. The results showed that the concentration of IFN-γ was significantly lower in the YM155 group than in the vehicle category (**Figure 6F**).

## Discussion

The present study used a mouse model of acute heart allograft rejection to demonstrate the protective effect of T-cell specific *Birc5* knockout and its underlying mechanism. In addition, the study used an animal model to explore the potential benefit of targeting survivin with YM155 in reducing acute allograft rejection. Previous studies showed that survivin regulates genes associated with the proliferation and development of hematopoietic stem cells and thymic T cells (13, 28). Additionally, survivin was reported to facilitate lymphocyte proliferation and promoted the maturation of T cells in arthritis (17). Given that *Birc5* has been shown to affect T-cell responses and that it increases during alloimmune responses, the present study further explored the role of *Birc5* in graft rejection.

Notably, Andersson et al. reduced the levels of survivin *in vivo* using a lentivirus shRNA and observed a significant increase in the levels of Tregs. Their results demonstrated that the more survivin was inhibited, the more Tregs were generated (17). Additionally, YM155 (A small molecule that inhibits survivin) may be used to inhibit survivin in *in vivo* and *in vitro* experiments. Notably, the antitumor activity of YM155 was first reported in a human malignant melanoma model and the molecule can also be applied in immune-related diseases (29). It was also reported that drugs could attenuate acute heart rejection by antagonizing the activity of key transcription factors that determine the differentiation of T cells subgroups (30). However, it is still unclear whether survivin limits the differentiation of alloreactive T cells to Tregs in an inflammation environment or only changes the flexibility of mature Tregs, leading to allotransplant rejection. The mechanisms underlying the effect of survivin are also unclear and should therefore be explored further.

In addition, previous studies showed that reduced the levels of survivin led to a decrease in the population of effector T cells in the spleen and local infiltration of T cells (17). Similarly, the present study showed that there was a significant decrease in the number of effector/effector memory T cells (CD62L^–^CD44^+^). There was also a decrease in the levels of IFN-γ in T cells in YM155 group, suggesting that a decrease in the number of effector/effector memory T cells is associated with an increase in the number of Tregs.

Moreover, a previous study showed that reducing the levels of survivin with YM155 in human adult T-cell lymphoma cells affected cell proliferation and induced cell death (31). Additionally, it was reported in HIV-1 patients, that resting CD4^+^ T cells with higher levels of *Birc5* mRNA had a long term proliferative ability while those treated with YM155 underwent more apoptosis and cell death (14). Furthermore, previous studies showed that *Birc5^-/-^* mice had impaired T cell proliferation and a reduced number but still with basically naturally phenotypes, consistent with the results obtained in the present study (13). In addition, the results herein revealed that knock out of *Birc5* suppressed the production of IFN-γ in T cells, similar to the results obtained from previous research (17).

The study also selected a safe dose of YM155 for *in vivo* and *in vitro* experiments, based on previous research (26, 32). The drug was administered through the intraperitoneal route and therefore not only affected T cells but also other cell population. According to previous research, over expression of survivin in grafts may suppress inflammation resulting from ischemia/reperfusion injury (33). However, the effects of survivin on host T cells are yet to be elucidated. Notably, survivin may have distinct functions in different cells, at various stages and even in different subcellular localizations. In cardiomyocytes for example, it inhibits apoptosis by reducing the expression of active Caspase-3, leading to less damage in the inception phase (34). In addition, previous research revealed that survivin inhibits apoptosis by maintaining mitochondrial integrity and associating with many molecules in the mitochondria (35). Up-regulation of survivin was also shown to confer protection from anthracycline-induced cardiotoxicity (36). Additionally, inhibition of caspases may have an effect on the microvascular endothelial cells in allografts, subsequently alleviating cardiac rejection (37). In the present study, the results showed that the YM155-induced cell apoptosis *in vitro* was partly mediated by activation of caspases and could be reversed upon the addition of a specific Caspase 3 Inhibitor. Moreover, PBMCs from healthy humans were treated with YM155 and similar results were obtained. The findings also showed that targeting survivin with YM155 could reduce the secretion of IFN-γ in human T lymphocytes.

In summary, the present study reported for the first time the effect of *Birc5* knockout on the survival of cardiac allografts. The results suggested that regulation of the caspase pathway may be partially responsible for the immune alterations that prolonged graft survival. Moreover, interfering with the expression of survivin using YM155 may be an effective strategy to inhibit rejection after transplantation.

## Data Availability Statement

The original contributions presented in the study are included in the article/**Supplementary Material**. Further inquiries can be directed to the corresponding authors.

## Ethics Statement

The animal study was reviewed and approved by the Institutional Animal Care and Use Committee of Tongji Medical College, Huazhong University of Science and Technology.

## Author Contributions

HX, JY, JX and JW conceived and designed the study. HX and JY carried out most of the experiments with help from ZC, YD, YZ. SL, JC, and LJ: feeding cage and identification mice. HX, JY, YL, and JC analyzed results and discussed the results. HX, JY and JW contributed to the writing of the manuscript. JX and JW coordinated this work, and all authors reviewed the manuscript. All authors contributed to the article and approved the submitted version.

## Funding

This work was supported by the National Natural Science Foundation of China (82071803, 81730015), Natural science fund of Hubei Province (2019AAA032) and the Fundamental Research Funds for the Central Universities (HUST No.2021GCRC037, No.2021yjsCXCY118, No. 2021yjsCXCY103).

## Conflict of Interest

The authors declare that the research was conducted in the absence of any commercial or financial relationships that could be construed as a potential conflict of interest.

## Publisher’s Note

All claims expressed in this article are solely those of the authors and do not necessarily represent those of their affiliated organizations, or those of the publisher, the editors and the reviewers. Any product that may be evaluated in this article, or claim that may be made by its manufacturer, is not guaranteed or endorsed by the publisher.

## Abbreviations

PBMCs, peripheral blood mononuclear cells; WT, wild‐type; MST, mean survival time; Treg, regulatory T cell; IFN-γ, interferon‐γ; i.p., intraperitoneal; FCM, flow cytometry; ELISA, enzyme-linked immunosorbent assay; MFI, mean fluorescence intensity.
