# Supplementary material for: Ablation of Survivin in T Cells Attenuates Acute Allograft Rejection after Murine Heterotopic Heart Transplantation by Inducing Apoptosis
Source: Front Immunol. 2021 Aug 6;12:710904. doi: 10.3389/fimmu.2021.710904 (PMC8377163; doi:10.3389/fimmu.2021.710904)
Supplement: Supplementary file 1 [file DataSheet_1.docx]

SUPPLEMENTAL EXPERIMENTAL PROCEDURES

**Immunohistochemistry**

Heart grafts, obtained on day 6 after transplantation, fixed in tissue fixing fluid (Servicebio G1101) for one day. Then they were paraffin-embedded and cut into sections stained by hematoxylin and eosin and captured by a conventional light microscope (original magnification 200×) as previously described(1). After performed background white balance, the captured sections images were graded by two investigators blinded for the groups independently using the recognized pathological criteria for following statistical analysis. Graft histology was evaluated by two experienced colleagues score independently and was quantified as follows. Heart cell infiltrates: 0, no cell infiltrate; 1, mild cell infiltrates; 2, medium cell infiltrates; 3, heavy cell infiltrates.

**Western blot analysis**

RIPA Lysis Buffer (Beyotime P0013) was used to lysed cultured cells on ice with Protease and Phosphatase Inhibitor Cocktail (Beyotime P1050) for 15 min. Caspase 3 Inhibitor Ac-DEVD-CHO (Beyotime C1206) Western blot was performed as protocol previously wrote(2). Survivin Monoclonal Antibody (J.33.5) (Thermo Fisher Scientific, MA5-15077), Anti-BIRC5 Antibody (BOSTER BM4334), Cleaved-CASP3 p17 (D175) Polyclonal Antibody (Elabscience E-AB-30004), Cleaved-PARP1 (D214) Polyclonal Antibody (Elabscience E-AB-30080), anti-GAPDH (Proteintech 60004-1-Ig) were used as primary antibodies. The intensity of bands was determined using ImageJ software.

**ELISA**

Concentration of IFN-γ in the supernatant of MLR was performed follow the instructions of Human IFN-gamma ELISA Kit (ABclonal Technology, WuHan, China).

**Flow cytometry**

For intracellular staining of cytokines, isolated cells were re-stimulated with 50 ng/ml phorbol 12-myristate 13-acetate (Sigma-Aldrich) and 0.5 μg/ml ionomycin (Sigma-Aldrich) in the company of GolgiStop (BD Biosciences) for 4 hours as protocol previously wrote(3). Later, Prepared cell suspensions were blocked with purified anti-mouse CD16/32 (Biolegend 101301) and then stained using the following fluorochrome-conjugated antibodies: CD4-PE (Biolegend 100407), CD4-APC/Cy7 (Biolegend 100526), CD8a-APC/Cy7 (BD Biosciences 557654), CD8-AlexaFluor700 (BD Bioscience 8229710), TCR-β-FITC (BD Biosciences 553170), CD62L-PE-Cy7 (BD Biosciences 560516), CD44-PerCP-Cy5.5 (BD Biosciences 560570), Foxp3-eFluor450 (eBioscience 48-5773-82), Foxp3-PE-Cy7 (eBioscience 25-5773-82), IFN-γ-PE-Cy7 (BD Biosciences 505825), Second antibody PE Donkey anti-rabbit IgG (Biolegend 406421), Annexin V-PE Apoptosis Detection Kit (Beyotime C1065L), and dead cells were excluded by Zombie Dyes (Biolegend 77143). Data were collected on LSRFortessa X-20 (BD Biosciences) and evaluated using Flowjo software (Tree Star).

**Statistical** **Analysis**

Cumulative graft survival was calculated using the Kaplan-Meier method. The statistical comparison was performed using the log-rank (Mantel-Cox) test. When appropriate, results were analyzed using the Mann-Whitney and Student *t*-tests. A *P* value less than 0.05 was considered significant.

**FIGURE LEGENDS OF SUPPLEMENTAL FIGURE**

**Supplemental Figure 1. YM155 similarly increase T cell apoptosis after activation in vitro, and can attenuate by apoptosis pathway inhibitor Ac-DEVD-CHO.**

(A) Representative histogram plots of Annexin V positive, follow bar graphs (B) of percent of their parent gated cells, T cells isolated from the spleens of WT mice following stimulated with anti-CD3/CD28 for 48h *in vitro* and then treated in the absence or presence of Ac-DEVD-CHO for 24h. Data are shown as mean values ± SD of one representative experiment out of three, * *p* < 0.05 , ** *p* < 0.01, ns: not statistically significant, n = 5 per group.

**Supplemental Figure 2.** **Survivin was upregulated in activated T cells and could be inhibited by YM155.**

(A)Western blot analysis of survivin expression at different time points. The CD4^+^ T cells were sorted following stimulation with anti-CD3/CD28 for 48h *in vitro*. (B) Graphs showing the quantitative analysis of survivin based on data from three repeated experiments. (C) CD4^+^ T cells from the spleen of WT or *Birc5^-/-^* mice were stimulated *in vitro* for 48h and the effect of *Birc5* knockout was verified through Western blot analysis. (D) Quantitative data from the results highlighted in (C). (E) The CD4^+^ T cells isolated from the spleen of WT mice were stimulated *in vitro* for 48h then treated with or without YM155 for 24h. The expression of survivin was examined through western blotting after stimulating the cells *in vitro*. GAPDH was used as an internal control. (F) Quantitative data from the results highlighted in (E). Data is presented as the mean ± SD of one representative experiment out of the three conducted, **p*<0.05, ***p* < 0.01.

**Supplemental Figure 3.** **Ablation *Birc5* reduce the on the effector** **subsets of** CD4+ **T cells in the spleen after heart transplantation.**

(A) The gating strategy in spleen. Leukocytes were gated according to morphology (SSC-A vs. FSC-A), doublets were excluded (FSC-H vs. FSC-A) and live immune cells were selected using Zombie Dyes. This gating strategy was used as a base for the following gates. CD4 vs. CD8 plot was used to discriminate between CD4^+^ and CD8^+^ T cells. (B) Representative flow plots of CD44 and CD62L expression on CD4^+^ T cells. (C) Bar graphs are shown with percentages ± SD of each gated sub-population of naive (CD62L^+^CD44^–^), effector/effector memory (CD62L^–^CD44^+^) and central memory (CD62L^+^CD44^+^) T cells on CD4^+^ T cells from indicated groups. (D) Percentage of CD4^+^ T cells and CD8^+^ T cells in spleen from indicated groups. (E) Absolute cell numbers of CD4^+^ T cells and CD8^+^ T cells in spleen from indicated groups. (F) Absolute cell numbers of CD4^+^Foxp3^+^ T cells, CD4^+^IFN-γ^+^ T cells and CD8^+^IFN-γ^+^ T cells in spleen from indicated groups. Data are representative of 3 independent experiments (n = 5 mice per group). Bars represent the mean ± SD. **p* < 0.05, ** *p* < 0.01, ns: not statistically significant.

1. Xiang M, Luo H, Wu J, Ren L, Ding X, Wu C, et al. ADAM23 in Cardiomyocyte Inhibits Cardiac Hypertrophy by Targeting FAK - AKT Signaling. J Am Heart Assoc. 2018;7(18):e008604.10.1161/JAHA.118.008604

2. Zhang A, Wang K, Zhou C, Gan Z, Ma D, Ye P, et al. Knockout of microRNA-155 ameliorates the Th1/Th17 immune response and tissue injury in chronic rejection. J Heart Lung Transplant. 2017;36(2):175-84.10.1016/j.healun.2016.04.018

3. Wu J, Zhang H, Shi X, Xiao X, Fan Y, Minze LJ, et al. Ablation of Transcription Factor IRF4 Promotes Transplant Acceptance by Driving Allogenic CD4(+) T Cell Dysfunction. Immunity. 2017;47(6):1114-28 e6.10.1016/j.immuni.2017.11.003
