# Supplementary figures and images for: Ablation of Survivin in T Cells Attenuates Acute Allograft Rejection after Murine Heterotopic Heart Transplantation by Inducing Apoptosis
Source: Front Immunol. 2021 Aug 6;12:710904. doi: 10.3389/fimmu.2021.710904 (PMC8377163; doi:10.3389/fimmu.2021.710904)

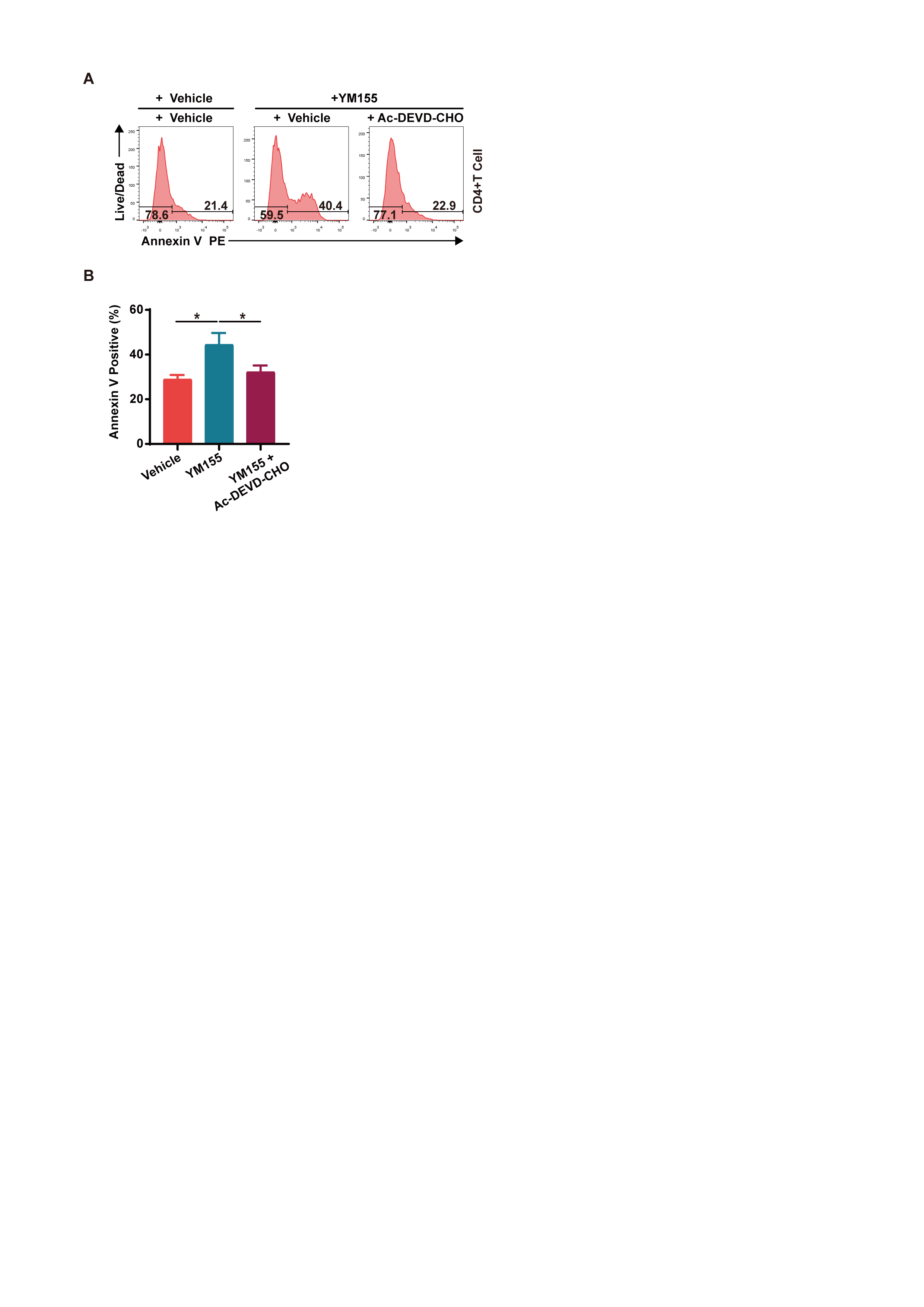

Supplement: Supplementary file 2 [file Image_1.tif]

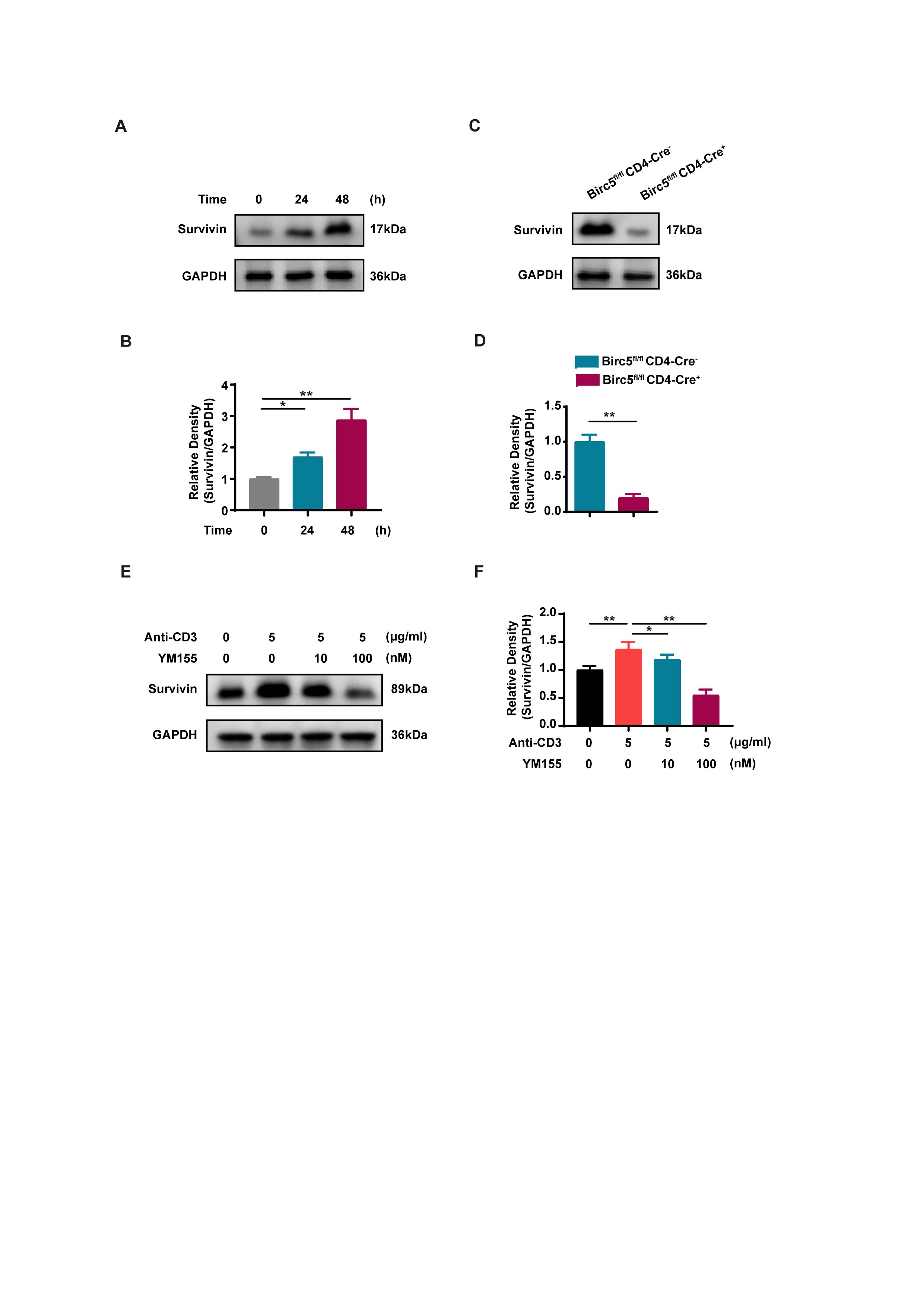

Supplement: Supplementary file 3 [file Image_2.tif]

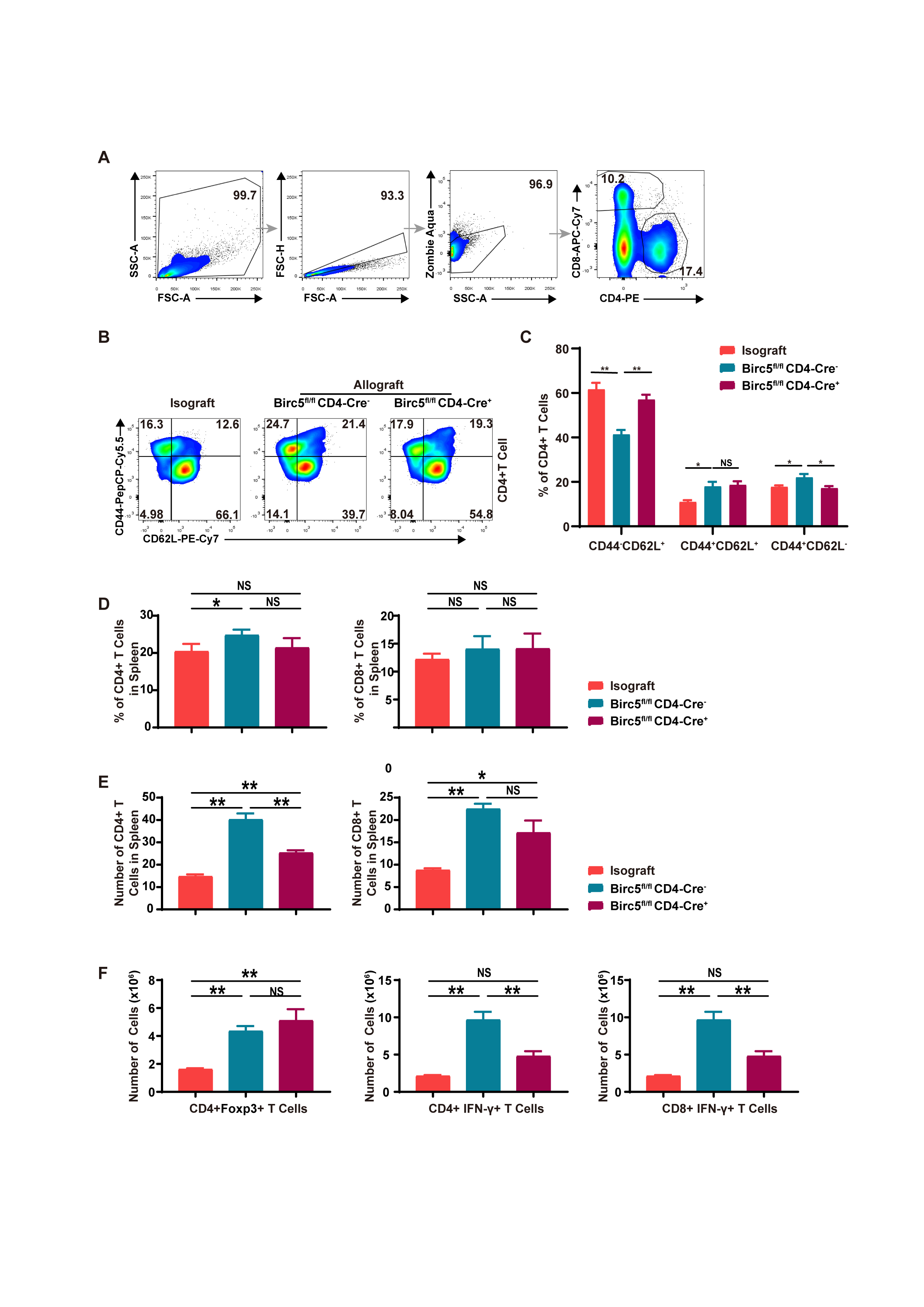

Supplement: Supplementary file 4 [file Image_3.tif]
